# Imorin: a sexual attractiveness pheromone in female red-bellied newts (*Cynops pyrrhogaster*)

**DOI:** 10.1038/srep41334

**Published:** 2017-01-25

**Authors:** Tomoaki Nakada, Fumiyo Toyoda, Kouhei Matsuda, Takashi Nakakura, Itaru Hasunuma, Kazutoshi Yamamoto, Satomi Onoue, Makoto Yokosuka, Sakae Kikuyama

**Affiliations:** 1 Department of Veterinary Medicine, Faculty of Veterinary Medicine, Nippon Veterinary and Life Science University, Tokyo 180-8602, Japan; 2 Department of Physiology, Nara Medical University, Nara 634-8521, Japan; 3 Laboratory of Regulatory Biology, Graduate School of Science and Engineering, University of Toyama, Toyama 930-8555, Japan; 4 Department of Anatomy and Cell Biology, Teikyo University School of Medicine, Tokyo 173-8605, Japan; 5 Department of Biology, Faculty of Science, Toho University, Chiba 274-8510, Japan; 6 Department of Biology, Faculty of Education and Integrated Sciences, Waseda University, Tokyo 169-8050, Japan; 7 Department of Pharmacokinetics and Pharmacodynamics, School of Pharmaceutical Sciences, University of Shizuoka, Shizuoka 422-8526, Japan

## Abstract

The male red-bellied newt (*Cynops pyrrhogaster*) approaches the female’s cloaca prior to performing any courtship behaviour, as if he is using some released substance to gauge whether she is sexually receptive. Therefore, we investigated whether such a female sexual attractiveness pheromone exists. We found that a tripeptide with amino acid sequence Ala-Glu-Phe is secreted by the ciliary cells in the epithelium of the proximal portion of the oviduct of sexually developed newts and confirmed that this is the major active substance in water in which sexually developed female newts have been kept. This substance only attracted sexually developed male newts and acted by stimulating the vomeronasal epithelial cells. This is the first female sexual attractiveness peptide pheromone to be identified in a vertebrate.

Most urodele amphibians reproduce by internal fertilization. However, the males of these species have no specific copulatory organs; therefore, they exhibit unique courtship behaviours to transfer sperm into the female’s cloaca^1^. It is believed that the male emits sex pheromones during courtship, which play an important role in keeping the female partner attracted to him until the reproductive behaviour has been successfully completed. Consequently, attempts have been made to identify the putative male pheromone that attracts sexually developed females. We previously isolated an active substance that is composed of 10-amino-acid residues from the abdominal gland–a sexually dimorphic bilobal gland extending from the cloaca to the ventral body cavity–of male red-bellied newts (*Cynops pyrrhogaster*)^2^. This peptide was named sodefrin and was both the first pheromone to be identified in an amphibian and the first peptide pheromone to be identified in a vertebrate. Since then, peptide and protein sex pheromones have been identified in several species of urodeles^3^,^4^,^5^,^6^,^7^, including geographical variants^8^. However, these pheromones have only been found in males.

In *Cynops* newts, the sexually developed male approaches the female with his snout directed toward her cloaca prior to commencing any courtship behaviour. This suggests that the male may recognise the female as being sexually receptive by means of some substance(s) released from her cloaca^9^. It has previously been confirmed that sexually developed *C. pyrrhogaster* males prefer water in which sexually developed females have been kept (i.e. female-conditioned water) and that the removal of the oviduct from sexually developed females attenuates this effect^10^. Therefore, we hypothesised that reproductive female newts emit a water-soluble substance to attract sexually developed males. In this study, we conducted experiments to identify and characterise this putative female pheromone and to confirm that it is the major active substance that occurs in female-conditioned water. We also examined the localization of this putative pheromone in the oviduct, as well as the responsiveness of the male olfactory organ to this substance.

## Results and Discussion

### Isolation and characterization of the female sexual attractiveness pheromone

**Figure 1:**
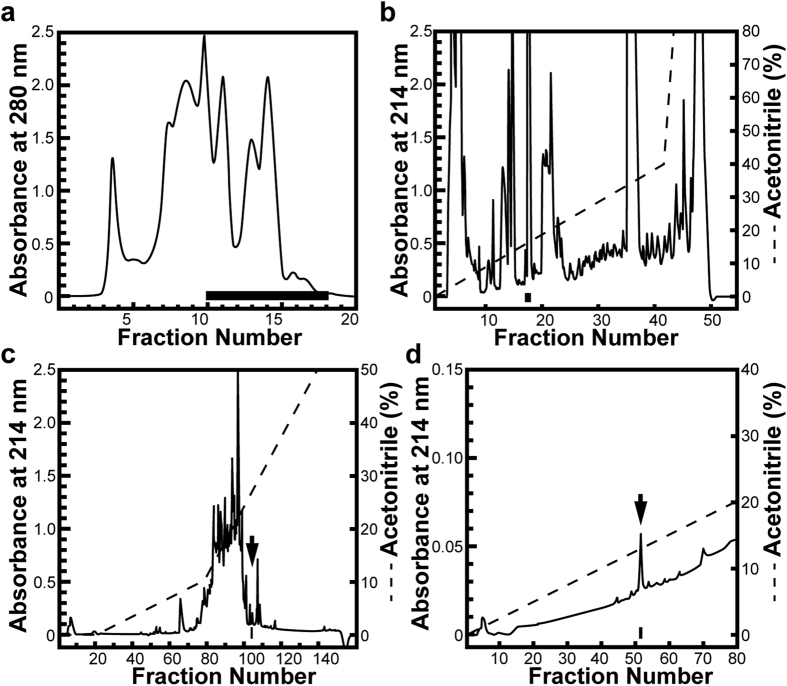
Representative profiles from the four chromatographic steps for purifying the male-attracting substance from sexually developed red-bellied newt (*Cynops pyrrhogaster*) oviducts. (**a**) Gel-filtration fast protein liquid chromatography (FPLC; Superose 12 HR 10/30) of the aqueous extract from 20 pieces of oviduct following partial purification on a Sep-Pak C_18_ cartridge. The low-molecular-weight (M.W. < 3,000) fractions denoted by the bar contained the active substance and were pooled for further purification. (**b**) Reversed phase high-performance liquid chromatography (HPLC; Puresil C18) of fractions containing the active substance (20 pieces equivalent) that were obtained in (**a**). The fractions that possessed a male-attracting activity are indicated by the bar. (**c**) Reversed phase HPLC (Nucleosil 120 3C18) of the fraction (105 pieces equivalent) possessing male-attracting activity that was separated in (**b**). The fraction that possessed a male-attracting activity is indicated by an arrow. (**d**) Reversed phase HPLC (Inertsil Ph) of the fraction (190 pieces equivalent) containing the male-attracting substance that was separated in (**c**). The peak of the fraction that possessed the male-attracting activity is indicated by an arrow. The final product was estimated to be 40 ng/piece. See Supplementary Fig. S2 for results of the associated preference tests.

Preference tests^10^ using sexually developed male newts showed that an aqueous extract of oviduct homogenate served as a male attractant. The amount equivalent to 1% of the oviducal content in a sponge block placed in a container filled with 3,000 mL of water was revealed to be enough to attract the male test animals (see Supplementary Fig. S1). Gel-filtration column chromatography (Fig. 1a) showed that the active component of this extract was contained in a fraction that had a relative molecular mass of < 3,000 (see Supplementary Fig. S2). To isolate the active substance from this fraction, we performed three cycles of reversed-phase high-performance liquid chromatography (HPLC) (Fig. 1b–d) and used each of the possible fractions in preference tests (see Supplementary Fig. S2), which resulted in the final product being obtained as a single chromatographic peak (Fig. 1d). Direct sequencing of this final product by automated Edman degradation revealed that it was a peptide with the amino acid sequence either Ala-Glu-Phe or Ala-Glu-Phe-Asp (see Supplementary Table S1). The retention time of the native peptide corresponded well with that of the synthetic Ala-Glu-Phe peptide, but not with that of the synthetic Ala-Glu-Phe-Asp. On the other hand, the retention time of the C-terminal amidated tripeptide did not correspond with that of the native peptide (see Supplementary Fig. S3). These results indicate that the final product is a tripeptide with amino acid sequence Ala-Glu-Phe and its C-terminus comprises a free phenylalanine residue. Furthermore, matrix-assisted laser desorption/ionization time-of-flight mass spectrometry (MALDI-TOF MS) analysis of an oviducal sample in which Ala-Glu-Phe molecules are expected to be contained, revealed the existence of a substance with a molecular mass that coincided with that of Ala-Glu-Phe (see Supplementary Fig. S4).

This peptide showed no sequence homology with any previously identified peptide^11^. Therefore, this male-attracting pheromone or female sexual attractiveness pheromone was designated ‘imorin’ (áimɔrin; derived from ‘imo,’ which is an ancient Japanese word meaning ‘beloved woman’ and ‘rin’ from sodefrin, a sex pheromone of the conspecific male newt^2^).

### Male-attracting activity of imorin

**Figure 2:**
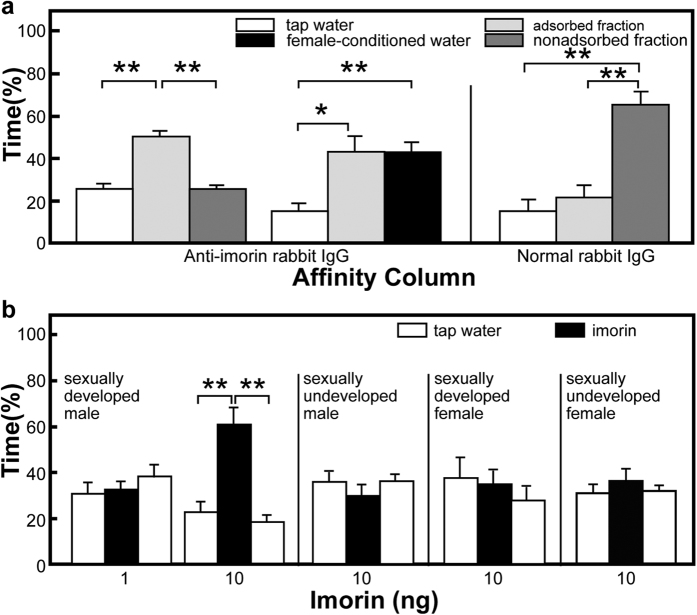
Evaluation of imorin activity. **(a)** Male-attracting activity of female-conditioned water subjected to an anti-imorin rabbit IgG- or normal rabbit IgG-coupled affinity column. The amount of sample used in each test was equivalent to one-tenth of the water conditioned by a single female. Sexually developed male red-bellied newts (*Cynops pyrrhogaster*) were used as test animals. **(b)** Attracting activity of imorin to sexually developed and undeveloped male and female newts. For the preference test, one sponge block contained imorin while the other two contained tap water. Results represent the mean values (±SEM) of eight tests. *P < 0.05, **P < 0.01 (Friedman’s two-way analysis of variance, followed by the Wilcoxon matched-pairs signed-ranks test).

The preference tests revealed that one-tenth of the water in which a single sexually developed female newt had been kept was sufficient to attract the male test animals (see Supplementary Fig. S5). To confirm that the male-attracting activity of the female-conditioned water was caused by imorin, we subjected the water to an anti-imorin rabbit IgG affinity column. Preference tests revealed that the non-adsorbed fraction that came through the antibody column possessed virtually no male-attracting activity, whereas the adsorbed fraction retained this activity with a potency equal to that of the original female-conditioned water (Fig. 2a). By contrast, the non-adsorbed fraction that came through the normal rabbit IgG column possessed a potent male-attracting activity, while the adsorbed faction had no male-attracting activity. Thus, it was demonstrated that imorin is the major male-attracting substance that is released by sexually developed females into the water environment.

Preference tests showed that 10 ng but not 1 ng imorin absorbed on a sponge block was sufficient to attract sexually developed male newts (Fig. 2b). By contrast, similar tests with sexually undeveloped males and sexually developed and undeveloped females did not yield a positive response to 10 ng imorin on a sponge block. These results indicate that behavioural responsiveness to imorin is dependent on reproductive state and sex of the newt.

### Responsiveness of vomeronasal cells to imorin

**Figure 3:**
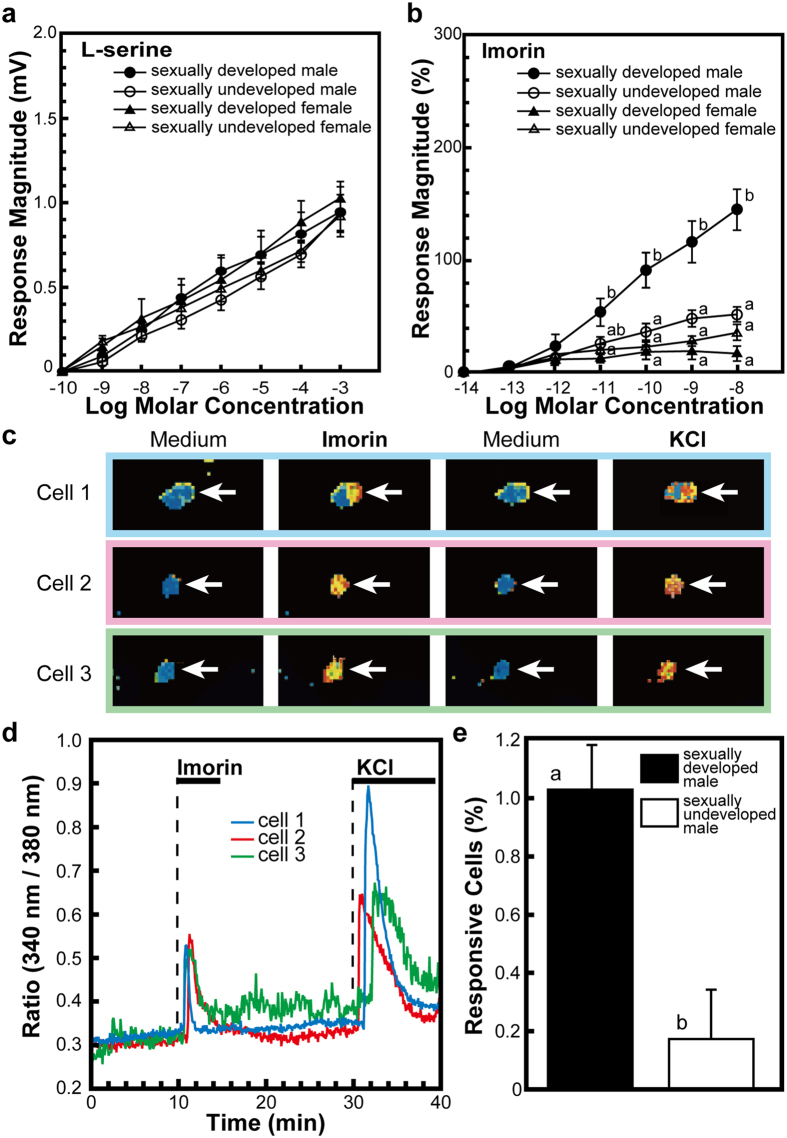
Responsiveness of the vomeronasal cells of male red-bellied newts (*Cynops pyrrhogaster*) to imorin. **(a)** Magnitude of the electro-olfactogram (EOG) response of the vomeronasal (VN) cells to L-serine (standard solution). Each point represents the mean (±SEM) of nine determinations. **(b)** Magnitude of the EOG response of the VN cells to imorin, expressed as a percentage of the response induced by 10^−5^ M L-serine as indicated in (a). Each point represents the mean (±SEM) of 8–9 determinations. Values with different superscript letters significantly differed from each other at the 5% level (Kruskal-Wallis one-way analysis of variance followed by Tukey’s honest significant difference test). **(c)** Changes in [Ca^2+^]_i_ in representative imorin-responsive cells from the VN epithelium of a sexually developed male newt. These cells responded to 10^−8^ M imorin and their viability was confirmed by an extracellular high-potassium stimulus (150 mM KCl). **(d)** Representative profiles of the changes in [Ca^2+^]_i_ expressed as the ratio of fluorescence intensity excited at 340 nm and 380 nm in the cells shown in (c). **(e)** Responses of VN epithelial cells from sexually developed and undeveloped newts to imorin (10^−8^ M). Each column represents the mean (±SEM) of 6–8 measurements of different samples. Values were significantly different from each other at the 5% level (Student’s *t-*test).

The olfactory system of urodeles consists of two morphologically distinct epithelia: the main olfactory epithelium and the vomeronasal (VN) epithelium^12^. The VN epithelium of the red-bellied newt lines a diverticulum that is situated lateral to the main chamber of the nasal cavity. It has traditionally been believed that social cues, including pheromonal signals, are detected by the VN epithelium, whereas conventional odours are detected by the main olfactory epithelium^13^,^14^. Therefore, the sensitivity of VN cells to imorin was examined electrophysiologically. First, we recorded electro-olfactograms (EOGs) from the epithelium of the VN organ using L-serine as a standard stimulant, which showed that the VN epithelia of sexually developed and undeveloped newts of both sexes invariably responded to L-serine to a similar extent in a concentration-dependent manner (Fig. 3a). We then recorded the EOG responses to imorin from the VN epithelia of sexually developed and undeveloped male and female newts, expressing each value as a percentage of the response to the 10^−5^ M L-serine standard. As a result, it was found that imorin markedly enhanced the EOG response in sexually developed male newts in a concentration-dependent manner, but only induced a moderate or low EOG response in sexually undeveloped males and sexually developed and undeveloped females. The magnitude of the response of the VN epithelium was significantly higher in sexually developed males than in sexually undeveloped males when subjected to 100 pM or higher concentrations of imorin (Fig. 3b).

The VN organ consists of several types of neurons that express distinct chemosensory receptors coupled with specific G proteins that play essential roles in signal transduction^15^,^16^,^17^. Pheromonal stimulation is believed to trigger these receptors, which mainly activate the phospholipase C pathway. This then results in the production of inositol 1,4,5-triphosphate and diacylglycerol, leading to an elevation of intracellular concentrations of Ca^2+^([Ca^2+^]_i_) and excitation of the receptor cells^17^,^18^,^19^,^20^. We have previously observed that the neurons in the VN organ of the red-bellied newt mostly express Gα_o_, but that quite a few neurons express Gα_olf_^21^. Therefore, we used a calcium imaging system to test whether imorin could increase [Ca^2+^]_i_ in dissociated VN cells from male newts. Imorin exposure resulted in an increase in [Ca^2+^]_i_ in a population of vomeronasal cells from sexually developed male newts (Fig. 3c and d). Furthermore, VN cells obtained from sexually developed male newts contained a significantly larger number of imorin-responsive cells than those from sexually undeveloped males (Fig. 3e).

In this experiment, a relatively small population of VN cells from sexually developed male newts responded to imorin through an elevation in [Ca^2+^]_i_. A similar phenomenon was also observed when VN cells from sexually developed female newts were subjected to the male sex pheromone sodefrin^22^. In mammals, VN cells express only one or a few of the large number of receptor types^23^,^24^, indicating that different neurons are sensitive to different ligands^25^. These findings indicate that this may also be the case in amphibians.

The results obtained by EOG and Ca^+2^ imaging studies confirmed the dependence of the responsiveness to imorin on the reproductive state and sex of the animal at the cellular level as well.

### Localization of imorin in the oviduct

**Figure 4:**
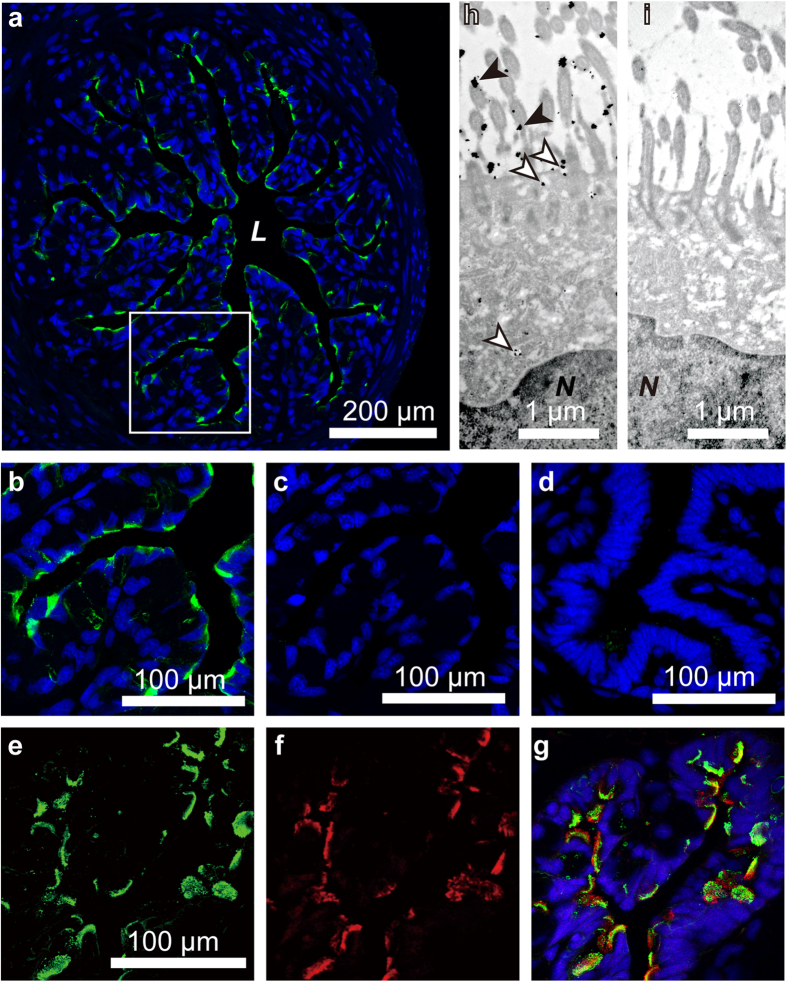
Localization of immunoreactive imorin in the oviduct. **(a)** The distribution of immunoreactive imorin (green) in the proximal portion of the oviduct of a sexually developed red-bellied newt (*Cynops pyrrhogaster*). Nuclei were stained with 4′,-6-diamidino-2-phenylindole (DAPI; blue). **(b)** Magnified image of the framed area in panel (a). (c) The oviducal section adjacent to the section shown in (b) stained with the anti-imorin antibody preabsorbed with imorin and counterstained with DAPI. **(d)** A section of the proximal portion of the oviduct of a sexually undeveloped newt that was stained with the imorin antibody and counterstained with DAPI. **(e** and **f**) A section of the proximal portion of the oviduct of a sexually developed newt that was double-labelled with anti-imorin antibody (green) and anti-acetylated α-tubulin antibody (red). **(g**) A merged image of imorin- and acetylated α-tubulin-immunoreactive cells, counterstained with DAPI. **(h)** A transmission immunoelectron microscopic analysis of the proximal portion of the oviduct of a sexually developed female newt. Imorin-immunoreactive signals were observed mainly in the cytoplasm (outlined arrowheads) of the ciliated cell and on the surface of the cilia (arrowheads). Note that the immunogold particles in the cytoplasm are enveloped in the secretory vesicles. **(i)** An ultra-thin section of the oviducal portion that is comparable to that shown in (h) stained with anti-imorin antibody preabsorbed with antigen. *L*, lumen; *N*, nucleus.

To determine the localization of imorin, oviducal sections were stained with antibodies against this peptide. Imorin-positive signals were detected on the apical side of the epithelial cells distributed in the proximal region of the oviducts of sexually developed females (Fig. 4a–c). By contrast, no imorin signals were detected in the epithelial cells in other regions of the oviduct or in the comparable region of the oviduct in sexually undeveloped females (Fig. 4d). We previously studied the effects of prolactin and sex steroids on the structural development and secretion of jelly substances in the red-bellied newt oviduct. This showed that a combination of prolactin and oestrogen stimulates the synthesis of jelly substances in the distal and middle portions of the oviduct, but not in the proximal portion, whereas it induces full structural development in the overall length of the oviduct^26^. The findings of the present experiment clearly indicate that at least one of the roles of the proximal portion of the oviduct is to secrete a male-attracting pheromonal substance.

Interestingly, the majority of the imorin signals corresponded well with the acetylated α-tubulin signals (Fig. 4e–g), indicating that it is the ciliated cells that are producing imorin. This was further supported by an immunoelectron microscopic study, which revealed that the immunogold particles indicating the presence of imorin were located within the secretory vesicles in the cytoplasm of ciliated cells and on the surface of the cilia extending into the lumen (Fig. 4h and i). We previously performed an immunoelectron microscopic study on the localization of sodefrin in the abdominal gland of sexually developed male newts, which showed that abundant immunogold particles indicating the presence of sodefrin existed within the secretory granules in the epithelial cells^8^,^27^. In the case of imorin, however, the density of the immunogold particles packaged in the secretory vesicles in the oviducal ciliated cells was not always high (Fig. 4h). It appears, therefore, that imorin is secreted constitutively from the ciliated cells, constantly sending signals through the cloacal orifice toward sexually developed males. Thus, imorin, with its relatively small molecular size, may diffuse effectively to reach males who are searching for sexually developed females.

Since the imorin molecule is composed of only three amino acid residues, it is likely that it has a precursor protein. However, isolation of the cDNA that encodes this putative imorin precursor protein from the oviducal cDNA library on the basis of information from these three amino acids is likely to prove difficult.

To date, several different groups of pheromone compounds emitted by sexually developed vertebrate females have been reported to attract and stimulate sexually developed conspecific males. The most extensive studies have been done in teleosts. These compounds include steroids^28^,^29^,^30^, steroid metabolites^31^, amino acid^32^ and prostaglandins^30^,^33^. In the Korean salamander, *Hynobius leechii* that externally fertilizes its eggs, the female also utilizes prostaglandins as male-attracting pheromone^34^. In the garter snake (*Thamniphis sirtalisparietalis*) female, the male-attracting pheromone is composed of long-chain (C_29_-C_37_) saturated and monounsaturated methyl ketones^35^. To the best of our knowledge, imorin in the red-bellied female newt is the first peptide pheromone with a male-attracting activity to be identified in a vertebrate.

## Conclusion

In urodeles, sex pheromones that are emitted by sexually developed males have been shown to function as courtship pheromones. In the present study, we demonstrated the existence of a male-attracting or sexual attractiveness pheromone that is released by sexually developed female red-bellied newts, which seems to plays an important role in driving males to perform their courtship behaviours. The responsiveness of sexually developed males to this female pheromone, imorin was demonstrated not only at the organismal level, but also at the cellular level. This raises the possibility that imorin released by the female stimulates the VN organ of the male and finally triggers the discharge of a courtship pheromone, sodefrin from the abdominal gland by way of the central nervous system. As for this female pheromone, however, the identities of its precursor protein in the oviduct and its receptor in the VN organ remain to be elucidated.

## Methods

All animal experiments were performed in accordance with the approved protocols and the guidelines of the Ethical Committee for the Care and Use of Laboratory Animals at Nippon Veterinary and Life Science University, and of the Steering Committee for Animal Experimentation at Nara Medical University.

### Animals

Adult male and female red-bellied newts (*Cynops pyrrhogaster*) were used in all experiments. For isolation of the male-attracting substance from female oviducts, we captured sexually developed females in the field and used them immediately. For all other purposes, we used adult newts of both sexes that had been kept under laboratory conditions for 3–4 months and then treated with either human chorionic gonadotropin (25 IU) in combination with ovine prolactin (1 IU) every other day for 14 days to attain a state of sexual development^36^ or saline as a control.

### Isolation of the male-attracting substance

The oviducts from 250 newts were dissected out under anaesthesia with 0.01% ethyl 3-aminobenzoate methanesulfonate (MS222; Sigma-Aldrich). The combined tissues (165 g) were homogenised with distilled water and the homogenate was then centrifuged at 157,000 × *g* for 1 hour at 4 °C. The supernatant was lyophilised, dissolved in 0.1% trifluoroacetic acid (TFA; 500 mL) and applied to a Sep-Pak C_18_ reversed-phase cartridge (Waters). The adsorbed substances were eluted with 80% acetonitrile/distilled water containing 0.1% TFA. The lyophilised eluate containing 30 mg protein was dissolved in 0.15 M NH_4_HCO_3_ (6 mL) and divided into 10 aliquots. Each aliquot was chromatographed on a gel-filtration chromatography column (Superose 12 HR 10/30, 10 mm × 300 mm; GE Healthcare) equilibrated with 0.15 M NH_4_HCO_3_ (pH 8.0) at a flow rate of 0.2 mL/min. Fractions that possessed a male-attracting activity were then pooled, freeze-dried, dissolved in 0.1% TFA (1 mL) and chromatographed on a reversed-phase HPLC column (Puresil C18, 4.6 mm × 150 mm; Waters) with a gradient of acetonitrile (0–80%) containing 0.1% TFA at a flow rate of 1.0 mL/min.

Following these two steps of chromatographic separation, the fractions that possessed a male-attracting activity were again pooled, freeze-dried and processed for further analyses. The sample dissolved in 0.1% TFA (1 mL) was chromatographed at a flow rate of 1.0 mL/min on a Nucleosil 120 3C18 column (4 × 250 mm; Marcherey-Nagel) with a gradient of acetonitrile (0–50%) containing 0.1% TFA. As the final purification step, the fractions exhibiting the activity were pooled, freeze-dried, dissolved in 0.1% TFA (10 μL) and chromatographed on a phenyl column (Inertsil Ph-3, 4.6 × 150 mm; GL Science) at a flow rate of 1.0 mL/min with a linear gradient of acetonitrile (0–20%) containing 0.1% TFA.

### Identification and characterisation of the male-attracting peptide

Direct NH_2_-terminal sequencing of the isolated substance by automated Edman degradation was contracted to Apro Science Co. C-terminal amidated and non-amidated peptides were then synthesised by Peptide Institute, Inc. based on the possible amino acid sequences of the native peptide. All of these peptides were subjected to an Inertsil Ph column (4 × 125 mm; GL Science) connected to the Inertsil Ph guard column (4 × 10 mm; GL Science) and their retention times were compared.

Existence of the male-attracting peptide molecule in the oviduct was confirmed using an aliquot of the fraction with a relative molecular mass of <3,000 that was prepared for the purification of the peptide. The sample was introduced to the affinity spin column for the male-attracting peptide and the adsorbed fraction was recovered according to the procedures described elsewhere (see *Affinity chromatography* in Methods section). After desalting through a Sep-Pak C_18_ reversed phase cartridge, the sample (0.1 piece equivalent) was applied for mass spectrometry to Autofrex MALDI-TOF (Bruker) using a cyano-4-hydroxy cinnamic acid matrix. For comparison, the synthetic Ala-Glu-Phe peptide (0.5 μg) was also subjected to MALDI-TOF MS analysis.

### Test for male-attracting activity

Preference tests^10^ were conducted to assess the male-attracting activity of the oviducal fractions, female-conditioned water and synthetic male-attracting peptide. Briefly, a plastic container (37-cm diameter) that was divided into three sectors was filled with 3,000 mL tap water and a test animal in a stainless steel mesh cylinder (15-cm diameter) was placed in the centre. Then a sponge block (5.6 × 7.3 × 3.4 cm) was gently placed into each sector: one containing the test substance dissolved in 100 mL tap water, another containing a control substance in 100 mL water or blank water and the third containing the same volume of water. Thirty seconds after introducing the sponge blocks, we removed the inner cylinder and recorded the test animal for 10 min to assess the position of its snout and the time it spent with its snout in each sector. Eight animals were used for each test and each test animal was only used once across the entire experiment.

### Preparation of female-conditioned water

Eight sexually developed females were kept in a container filled with 800 mL tap water for 48 hours. The water was then filtered using Whatman filter paper and the filtrate was lyophilised. The lyophilised sample was dissolved in 50 mM Tris-HCl (8.5 mL) containing 150 mM NaCl (pH 7.5) and filtered again using 0.45-μm-pore filters (DISMIC-25CS, Advantec). One quarter of the total amount of water was saved while the remainder was used for affinity chromatography.

### Antibody against the male-attracting peptide

To develop an antiserum against the male-attracting substance, we immunised a white rabbit by injecting subcutaneously with synthetic male attracting peptide (100 μg) that was extended on its COOH-terminus with the hydrophilic linker, of which Cys residue was coupled to keyhole limpet haemocyanin, for 5 times during the period of 60 days. Following immunization, we harvested the serum from the blood of the immunised rabbit and isolated IgG from it using the ammonium sulphate precipitation method. This was then purified by affinity chromatography on a recombinant Protein A sepharose column.

### Affinity chromatography

An affinity batch spin column (3.2 mL) was prepared for the purified anti-male-attracting peptide rabbit IgG or purified normal rabbit IgG by mixing the IgG (32 mg) with Protein A-coupled Sepharose (Protein A HP SpinTrap; GE Healthcare), followed by covalent cross-linking of the bound antibody using dimethoxypropan. The equivalent amount to water conditioned by 2.5 females was dissolved in binding buffer (50 mM Tris-HCl, pH 7.5 containing 150 mM NaCl; 6.4 mL), introduced into the spin column, mixed by manual inversion and then incubated with slow end-over-end mixing for 1 hour. The non-adsorbed fraction was collected by centrifugation. The column was then washed five times with 50 mM Tris-HCl and 150 mM NaCl with 2 M urea at pH 7.5 (12.8 mL), and the adsorbed substances were eluted with 0.1 M glycine with 2 M urea at pH 2.9 (22.4 mL).

### Histological analyses

#### Immunohistochemistry

Sexually developed and undeveloped females were perfused with 4% paraformaldehyde (PFA) in 0.1 M phosphate buffer (PB; 5 mL). Their oviducts were then dissected out and post-fixed in 4% PFA in 0.1 M PB overnight, following which they were transferred into 20% sucrose/0.7 × phosphate-buffered saline (PBS; pH 7.4) overnight and embedded in OCT compound (Sakura Finetechnical) at −80 °C. For immunohistochemical analysis, coronal sections (12 μm-thickness) were rinsed with 0.7 × PBS containing 0.05% Triton X-100 and blocked with 10% Block Ace (Yukijirushi Milk Products Co.) for 30 min. The sections were then incubated with the anti-male-attracting peptide (1:2,000) and/or anti-acetylated α-tubulin antibody (1:1,000; T6793; Sigma-Aldrich) at 4 °C for 2 days. Goat anti-rabbit antibody conjugated with Alexa Fluor 488 (1:1,000; Invitrogen) and goat anti-mouse antibody conjugated with Alexa Fluor 594 (1:1,000; Invitrogen) were used as the respective secondary antibodies. Nuclear staining was performed with 4′,6-diamidino-2-phenylindole (DAPI).

#### Immunoelectron microscopy

Cell typing with immunoelectron microscopy was performed according to the method described previously^37^. Briefly, the cryosections of oviducts were washed in PBS containing 0.1% Triton X-100 (PBST), sequentially treated with the rabbit anti-male-attracting peptide (1:2,000) in PBST containing 1% bovine serum albumin (1% BSA/PBST) overnight at 4 °C and then washed again with PBST. The sections were then incubated with 1.4-nm nanogold particles conjugated to Fab fragments of anti-rabbit IgG diluted to 1:100 with 1% BSA/PBST for 2 hours. Following this, the sections were washed with PBST and then fixed with 1% glutaraldehyde for 10 min, washed thoroughly with water and incubated with silver enhancement solution (HQ-Silver kit; Nanoprobe). After a quick rinse with water, they were immersed in 0.05% aurochlorohydric acid solution for 2 min and then rinsed in water. The specimens were then fixed with osmium tetroxide in 0.1 M PB for 20 min, dehydrated with ethanol and embedded by inverting Epon/Araldite-filled gelatin capsules over the slide-attached tissue sections. The ultrathin sections were stained with uranyl acetate and lead citrate and then examined with a Hitachi H-7500 electron microscope at 80 kV.

### EOG recording

Once the newts had been anaesthetised with 0.1% MS222 and immobilised with an intramuscular injection of 0.03% *d-*tubocurarine chloride (0.05 mL), the ventral surface of the nasal epithelium was exposed. EOG responses were recorded at the surface of the VN epithelium using a gelatin-filled capillary glass pipette (80–100 μm tip diameter) bridged to an Ag-AgCl electrode filled with 3 M KCl. The position of this electrode was adjusted to yield both a maximal response to 10^−5^ M L-serine chosen as a standard stimulant^38^ and a minimal response to distilled water. A reference electrode of the same type was placed on the skin surface near the perfused naris and grounded this. Then constant volumes (0.25 mL) of aqueous stimulant solutions were injected into the olfactory cavity through the external nares at a constant rate (3 mL/min) using a microsyringe pump. The stimulus duration at the epithelium was 5 sec. Each stimulant solution was tested three times with a 2-min interval between tests to obtain an average response. The epithelium was rinsed with distilled water during each interval. EOG responses were amplified with a DC preamplifier, displayed on an oscilloscope and stored on a computer, following which they were analysed using the PowerLab two-channel physiological instrument system (PowerLab 2/20; ADInstruments). The magnitude of each EOG response was measured as the height from the baseline to the peak of each phasic displacement. Replicate readings were averaged and the blank water response was subtracted from the value. The magnitude of the EOG responses to the male-attracting peptide was then expressed as a percentage of the response to the 10^−5^ M L-serine standard.

### **Ca** ^**2+**^imaging

#### Preparation of newt VN cells

The epithelia of the VN organ from the newts were excised under anaesthesia and the mucosa was incubated for 7 min at 35 °C with collagenase (0.1 mg/mL) in HEPES-buffered standard solution (HBSS) containing no Mg^2+^/Ca^2+^and then physically dissociated by pipetting. The cell suspension was then filtrated using a 70-μm nylon membrane filter. Following centrifugation at 700 rpm, the isolated cells were washed and kept in HEPES-buffered M199 (Nissui Pharmaceutical Co., Ltd.). The isolated VN cells were plated on cover slips coated with Concanavalin A (Sigma-Aldrich) and maintained at 4 °C until use. Calcium imaging analysis began within 2 hours of the isolation of VN cells, and the cell viability and responsiveness of the cells to the test substance were confirmed to be stable.

#### Measurement of [Ca^2+^]

[Ca^2+^]_i_ was measured using the method described previously^22^. Briefly, the dissociated cells on coverslips were incubated with 4 μM fura-2 acetoxymethyl ester (fura-2-AM; Dojin Chemical) in M199 containing 5% FBS for 1 hour at 4 °C. The cells were then mounted in a chamber, placed on the stage of an inverted microscope and perfused with HBSS using a peristaltic pump at 0.5 mL/min. The cells were excited at 340 nm (F340) and 380 nm (F380), and the fura-2 fluorescence was detected every 5–8 sec by an intensified charge-coupled device camera. The F340/F380 ratio was calculated using the Argus-50 system (Hamamatsu Photonics). Changes in the ratio of approximately 100 cells loaded with fura-2-AM within a low power-field were monitored at a time. The monitoring was carried out 3–5 times for each set of 4–5 samples from different individuals.

The test substance was added into the chamber by a perfusion system following a stabilization period (>5 min) in HBSS, with a switch time of <1 sec between the different solutions. The baseline was recorded for 60 sec prior to each stimulus. According to the criterion adopted by Huang *et al*.^39^, the response was defined as positive when the peak value was at least twice the amplitude of the baseline fluctuation (mean + 2 SD). The viability of the cells was confirmed by subjecting them to an extracellular high-potassium stimulus. The ratio between the number of responsive cells and the total number of cells examined was expressed as the percent responsive cells.

### Statistical analyses

All quantitative results are presented as means ± SEM. Details of the statistical analyses are described in the relevant figure legends. A *P*-value of 0.05 or less was considered significant.

## Additional Information

**How to cite this article**: Nakada, T. *et al*. Imorin: a sexual attractiveness pheromone in female red-bellied newts (*Cynops pyrrhogaster*). *Sci. Rep.* **7**, 41334; doi: 10.1038/srep41334 (2017).

**Publisher's note:** Springer Nature remains neutral with regard to jurisdictional claims in published maps and institutional affiliations.

## Supplementary Material

Supplementary Materials
