# Supplementary material for: Imorin: a sexual attractiveness pheromone in female red-bellied newts (Cynops pyrrhogaster)
Source: Sci Rep. 2017 Jan 25;7:41334. doi: 10.1038/srep41334 (PMC5264602; doi:10.1038/srep41334)
Supplement: Supplementary Materials [file srep41334-s1.doc]

Supplementary Materials for

Imorin: a sexual attractiveness pheromone in female red-bellied newts (*Cynops pyrrhogaster*)

Tomoaki Nakada, Fumiyo Toyoda, Kouhei Matsuda, Takashi Nakakura, Itaru Hasunuma, Kazutoshi Yamamoto, Satomi Onoue, Makoto Yokosuka, Sakae Kikuyama.

Correspondence to: tnakada@nvlu.ac.jp and kick-yama@waseda.jp

**This file includes:**

Supplementary Figures S1 to S5 and Table S1


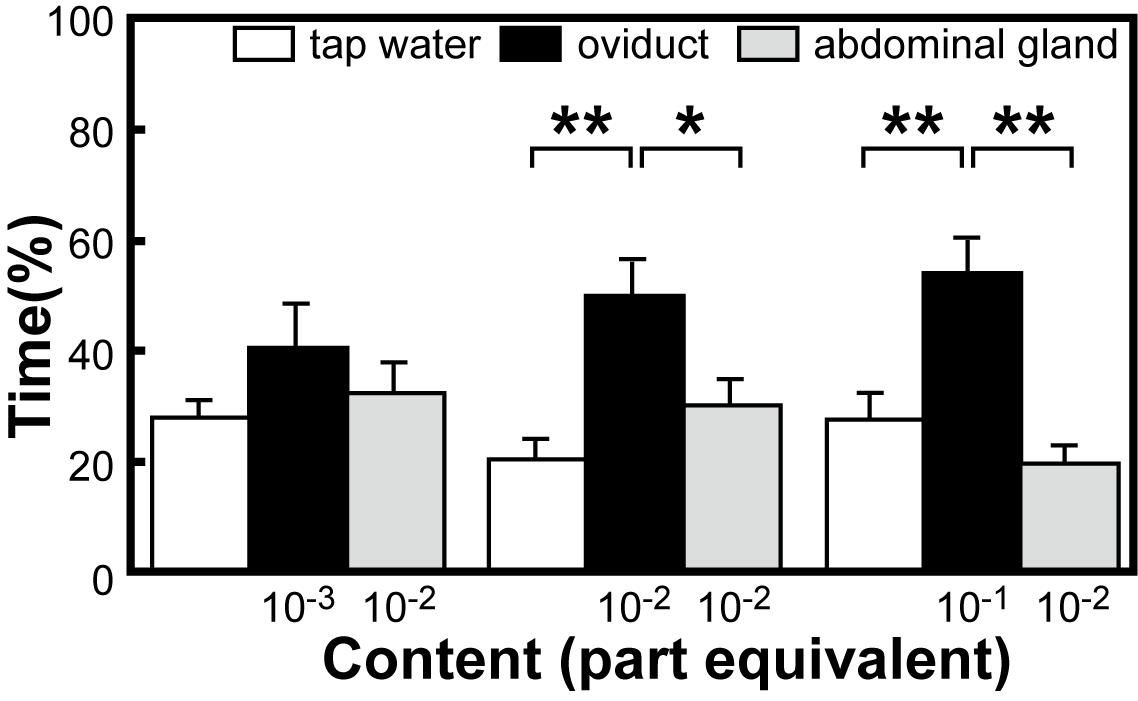


**Supplementary Figure S1.** **Male-attracting activity of the oviduct of sexually developed female red-bellied newts (*Cynops pyrrhogaster*).** Twenty oviducts were homogenized in distilled water (20 mL). The homogenate was centrifuged at 5,000 × *g* for 1 hour at 4°C, and the supernatant was then lyophilized and used as the test substance. Abdominal gland extract was prepared in a similar way and used as a control substance. Preference testing was performed as previously described^8^. Each sponge block contained either tap water or the indicated amount of extract from the oviduct or abdominal gland. Results represent the mean values (±SE) of eight tests. **P* < 0.05, ***P* < 0.02 (Friedman’s two-way analysis of variance, followed by the Wilcoxon matched-pairs signed-ranks test).


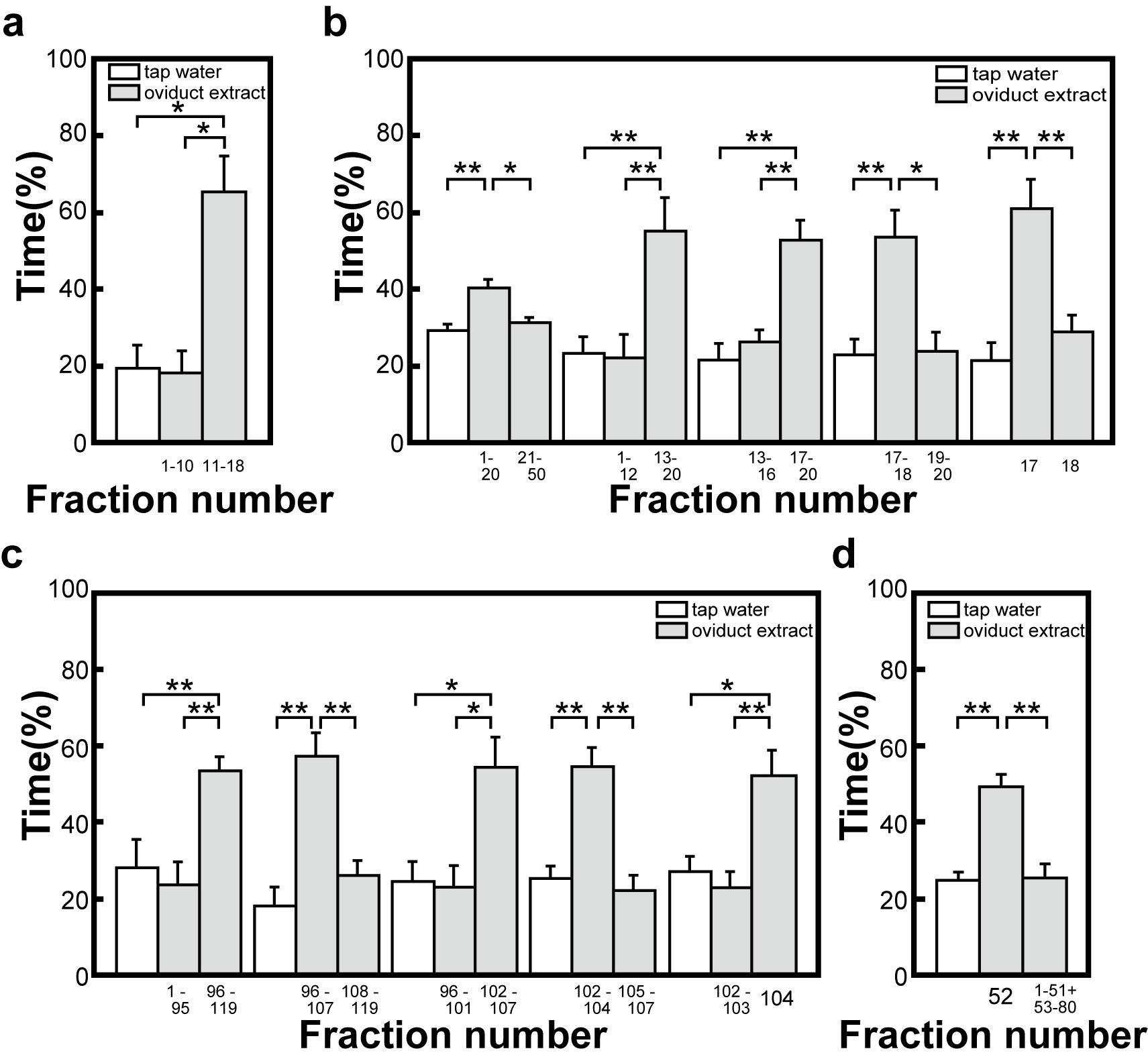


**Supplementary Figure S2. Preference tests using fractions obtained at each stage of purification of the male-attracting substance. (a)** Preference tests using fractions obtained by subjecting the aqueous extract of the oviducts of sexually developed female red-bellied newts (*Cynops pyrrhogaster*) to gel-filtration and fast protein liquid chromatography (FPLC). Each sponge block contained tap water or fractions from 0.1 part equivalent of the oviduct. **(b)** Preference tests using fractions obtained by subjecting the active substance obtained in (a) to reversed phase high-performance liquid chromatography (HPLC; Puresil C18). Each sponge block contained tap water or fractions from 0.1 part equivalent of the oviduct. **(c)** Preference tests using fractions (0.1 part equivalent) obtained by subjecting the active substance obtained in (b) to reversed phase HPLC (Nucleosil 120 3C18). Following five screening procedures, fraction 104 was revealed to contain the male-attracting substance. **(d)** Preference tests using fractions (0.1 part equivalent) obtained by subjecting the active substance obtained in (c) to reversed phase HPLC (Inertsil Ph). The single peak fraction (fraction 52) was found to possess a male-attracting activity. Results are expressed as the mean values (±SE) of eight tests (*n* = 8). Each test was performed using different individuals. **P* < 0.05, ***P* < 0.01 (Friedman's two-way analysis of variance, followed by the Wilcoxon matched-pairs signed-ranks test).


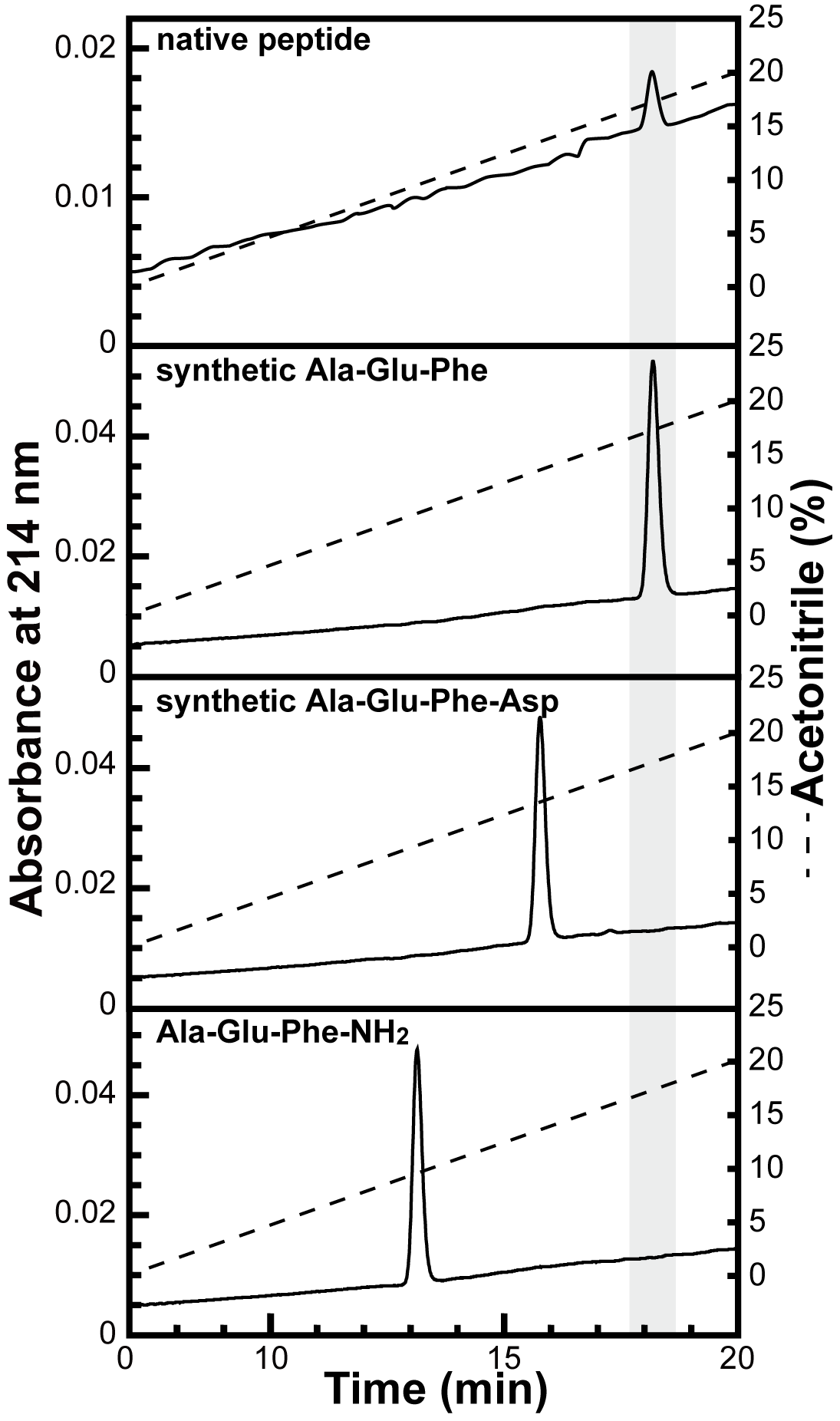


**Supplementary Figure S3. Comparison of retention times for the native peptide isolated from the oviduct of female red-bellied newts and synthetic Ala-Glu-Phe, Ala-Glu-Phe-Asp and Ala-Glu-Phe-NH_2_ peptides.** The native peptide (1 µg), and synthetic peptides (10 µg each) were subjected to chromatography using an Inertsil Ph column.

**
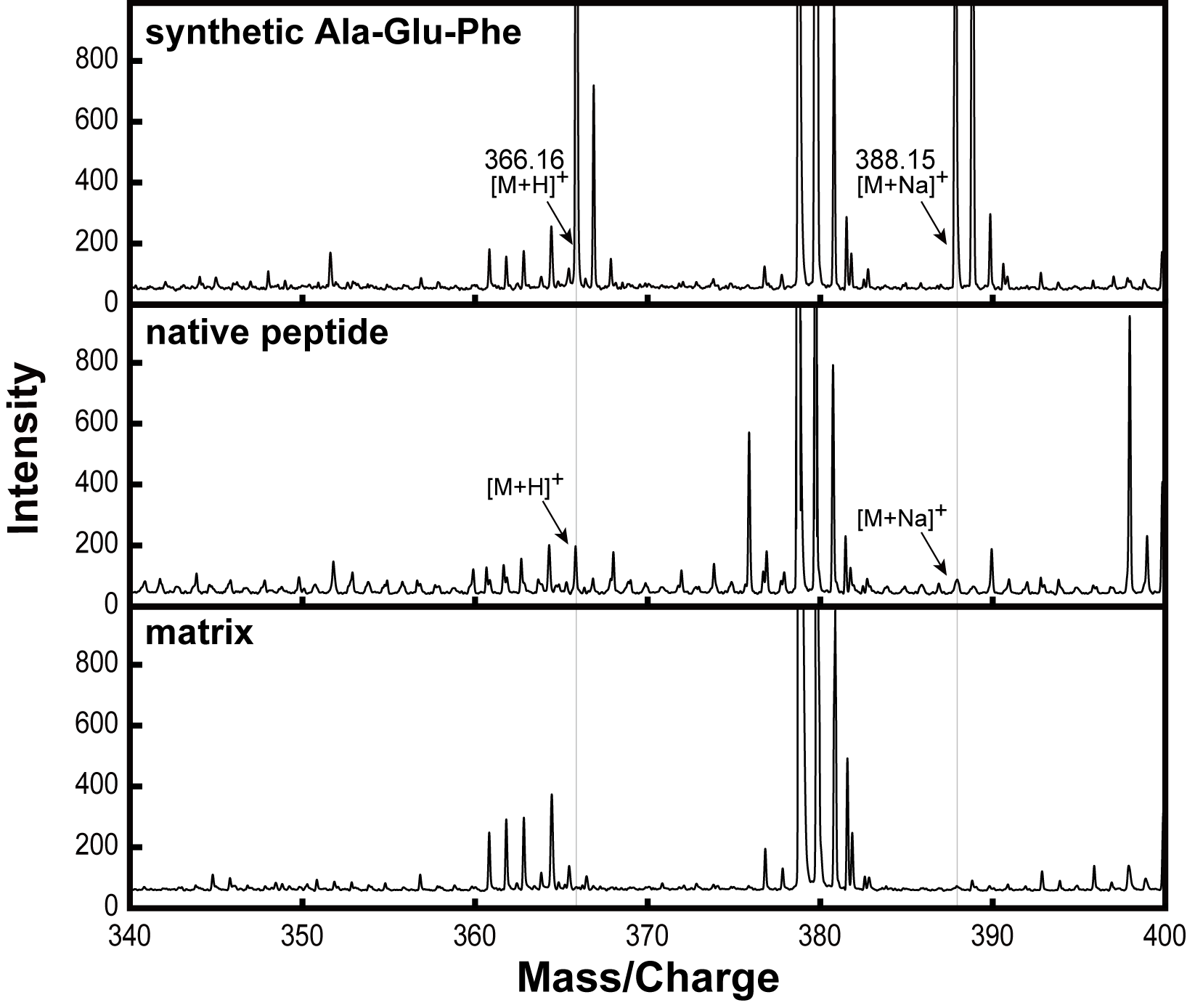
**

**Supplementary Figure S4. Comparison of MALDI-TOF spectra for the synthetic Ala-Glu-Phe and a native peptide from the oviduct.** The calculated monoisotopic molecular mass of the Ala-Glu-Phe is 365.16. Both the endogenous peptide and synthetic Ala-Glu-Phe appeared as [Ala-Glu-Phe+H]^+^ and [Ala-Glu-Phe+Na]^+^ with molecular masses of 366.16 and 388.15, respectively.

**
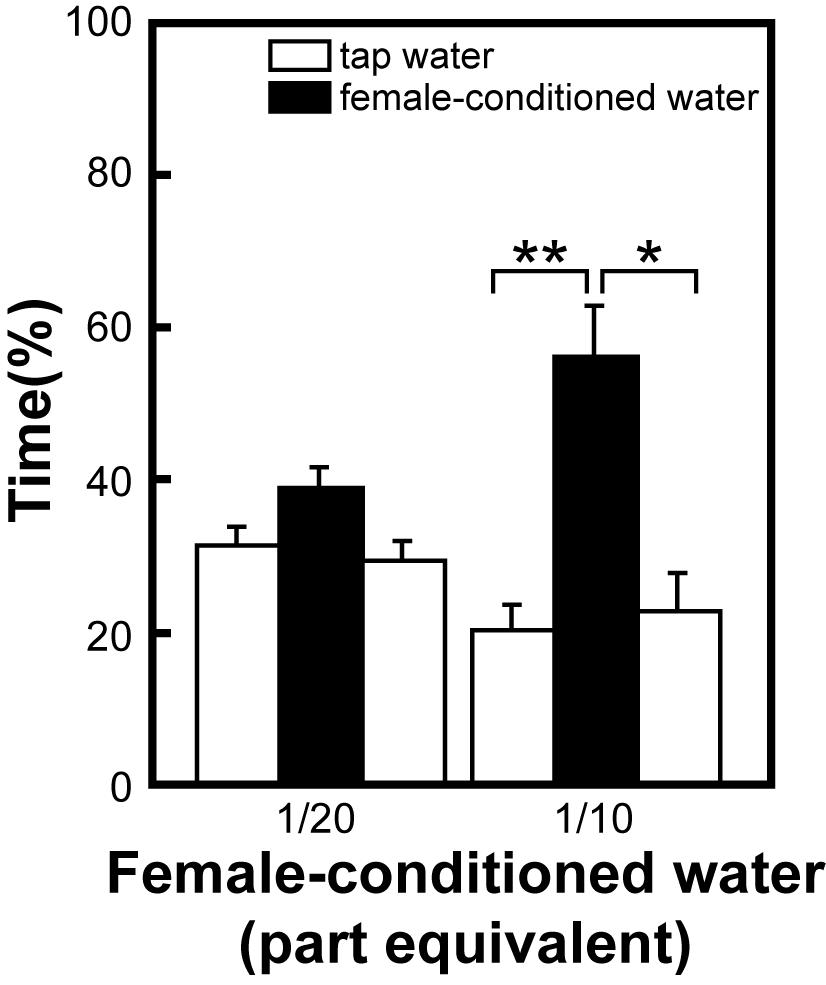
**

**Supplementary Figure S5. Male-attracting activity of the female-conditioned water.** The amounts of sample used in the preference tests were equivalent to one-twentieth and one-tenth of the water conditioned by a single sexually developed female red-bellied newt (*Cynops pyrrhogaster*). For the preference tests, one sponge block contained test substance and the other two contained tap water. Sexually developed male newts were used as test animals. Results represent the mean values (±SE) of eight tests. **P* < 0.05, ***P* < 0.02 (Friedman’s two-way analysis of variance, followed by the Wilcoxon matched-pairs signed-ranks test).

**Supplementary Table S1. Amino acid sequence analyses of the male-attracting substance.** Direct NH_2_-terminal sequencing of the final product was performed using automated Edman degradation. The substance obtained from 40 pieces of the oviducts was used for the analyses.

| Cycle　No. | major amino acid | pmol |
| --- | --- | --- |
| 1 | Ala | 41.2 |
| 2 | Glu | 48.8 |
| 3 | Phe | 19.5 |
| 4 | Asp | 8.0 |
